# Supplementary material for: Adherence to 24-hour integrated activity guidelines among infants, toddlers and preschool children in Singapore
Source: PLoS One. 2024 Feb 26;19(2):e0298968. doi: 10.1371/journal.pone.0298968 (PMC10896501; doi:10.1371/journal.pone.0298968)
Supplement: S1 Table — (DOCX) [file pone.0298968.s001.docx]

S1 Table: Parental practices of lifestyle behaviours by age group

|  | **Infants (n=219)** | **Toddlers (n=379)** | **Pre-schoolers (n=303)** | **p value** |
| --- | --- | --- | --- | --- |
|  | n (%) | n (%) | n (%) |  |
| **Being active with, or in front of child** |  |  |  | 0.001 |
| Never/Rarely | 8 (3.7) | 3 (0.8) | 18 (6.0) |  |
| Occasionally | 47 (21.7) | 84 (22.6) | 142 (47.7) |  |
| Frequently/all the time | 163 (75.1) | 284 (76.5) | 138 (46.3) |  |
| **Restraining child daily from free spontaneous movement** |  |  |  | 0.001 |
| Never/Rarely | 138 (63.0) | 274 (73.7) | 241 (80.9) |  |
| Occasionally | 45 (20.5) | 59 (15.9) | 36 (12.1) |  |
| Frequently/all the time | 36 (16.4) | 39 (10.5) | 21 (7.0) |  |
| **Ensuring child receives consistent bedtime routine and bedtime** |  |  |  | 0.104 |
| Never/Rarely | 5 (2.3) | 6 (1.6) | 6 (2.0) |  |
| Occasionally | 37 (16.9) | 40 (10.8) | 52 (17.4) |  |
| Frequently/all the time | 177 (80.8) | 324 (87.6) | 240 (80.5) |  |
| **Limiting own recreational screen time when spending time with child** |  |  |  | 0.001 |
| Never/Rarely | 77 (35.2) | 117 (31.4) | 21 (7.1) |  |
| Occasionally | 65 (29.7) | 113 (30.4) | 168 (56.8) |  |
| Frequently/all the time | 77 (35.2) | 142 (38.2) | 107 (36.1) |  |
| **Preparing or providing meals for child that are well-balanced and generally healthy.** |  |  |  | 0.001 |
| Never/Rarely | 22 (10.0) | 4 (1.1) | 22 (7.4) |  |
| Occasionally | 36 (16.4) | 62 (16.7) | 105 (35.5) |  |
| Frequently/all the time | 161 (73.5) | 306 (82.3) | 169 (57.1) |  |
| **Limiting own intake of unhealthy food and beverages.** |  |  |  | 0.001 |
| Never/Rarely | 87 (40.1) | 140 (37.7) | 24 (8.1) |  |
| Occasionally | 77 (35.5) | 121 (32.6) | 173 (58.4) |  |
| Frequently/all the time | 53 (24.4) | 110 (29.6) | 99 (33.4) |  |
| **Child has at least one meal with the family each day.** |  |  |  | 0.001 |
| Never/Rarely | 37 (16.9) | 17 (4.6) | 7 (2.4) |  |
| Occasionally | 27 (12.3) | 34 (9.2) | 23 (7.8) |  |
| Frequently/all the time | 155 (70.8) | 319 (86.2) | 266 (89.9) |  |

Missing data: Being active with, or in front of child - (n=1), toddlers (n=8), pre-schoolers (n=5); Restraining child daily from free spontaneous movement - (n=5), pre-schoolers (n=5); Ensuring child receives consistent bedtime routine and bedtime - Toddler (n=9), Pre-schoolers (n=8) ; Limiting own recreational screen time when spending time with child - Toddlers (n=7), Pre-schoolers (n=7) ; Preparing or providing meals for child that are well-balanced and generally healthy - Toddlers (n=7), Pre-schoolers (n=7) ;  Limiting own intake of unhealthy food and beverages - (n=2), Toddlers (n=8), Pre-schoolers (n=7) ; Child has at least one meal with the family each day. Toddler (n=9), Pre-schoolers (n=7).
