# Supplementary material for: Adherence to 24-hour integrated activity guidelines among infants, toddlers and preschool children in Singapore
Source: PLoS One. 2024 Feb 26;19(2):e0298968. doi: 10.1371/journal.pone.0298968 (PMC10896501; doi:10.1371/journal.pone.0298968)
Supplement: S2 Table — (DOCX) [file pone.0298968.s002.docx]

**S2 Table: Parental perceptions of child’s health by age group**

|  | Infants (n=219) | Toddlers (n=379) | Pre-schoolers (n=303) | p value |
| --- | --- | --- | --- | --- |
|  | n (%) | n (%) | n (%) |  |
| **Weight of child** |  |  |  | 0.003 |
| Overweight | 9 (4.1) | 13 (3.5) | 11 (3.7) |  |
| Normal weight | 194 (88.6) | 297 (79.8) | 238 (80.1) |  |
| Underweight | 13 (5.9) | 57 (15.3) | 33 (11.1) |  |
| Not sure | 3 (1.4) | 5 (1.3) | 14 (4.7) |  |
| **Child receiving adequate physical activity** |  |  |  | 0.001 |
| Yes | 115 (52.8) | 283 (76.3) | 156 (52.4) |  |
| No | 44 (20.2) | 45 (12.1) | 57 (19.2) |  |
| Not sure | 59 (27.1) | 43 (11.6) | 84 (28.3) |  |
| **Concerns over screen viewing time of child** |  |  |  | 0.001 |
| Yes | 38 (17.4) | 106 (28.5) | 161 (54.0) |  |
| No | 163 (74.4) | 189 (50.8) | 101 (33.9) |  |
| Not sure | 18 (8.2) | 77 (20.7) | 35 (11.7) |  |
| **Child receiving adequate sleep** |  |  |  | 0.001 |
| Yes | 184 (84.0) | 324 (87.1) | 231 (77.8) |  |
| No | 9 (4.1) | 30 (8.1) | 23 (7.7) |  |
| Not sure | 26 (11.9) | 18 (4.8) | 43 (14.5) |  |

Missing values: Weight of child - (toddler n=7), pre-schoolers (n=6); Child receiving adequate physical activity - Infant (n=1), Toddler (n=8), pre-schoolers (n=6); Concerns over screen viewing time of child; Toddler (n=7), Pre-schooler (n=6); Child receiving adequate sleep Toddler (n=7), Pre-schooler (n=7)
