# Supplementary material for: Adherence to 24-hour integrated activity guidelines among infants, toddlers and preschool children in Singapore
Source: PLoS One. 2024 Feb 26;19(2):e0298968. doi: 10.1371/journal.pone.0298968 (PMC10896501; doi:10.1371/journal.pone.0298968)
Supplement: S3 Table — (DOCX) [file pone.0298968.s003.docx]

S3 Table: Parental awareness of lifestyle behaviour guideline recommendations by age group

|  | Infants (n=219) | Toddlers (n=379) | Pre-schoolers (n=303) | p value |
| --- | --- | --- | --- | --- |
|  | n (%) | n (%) | n (%) |  |
| **Physical activity guidelines** |  |  |  | 0.001 |
| Not aware | 159 (72.6) | 217 (58.3) | 162 (54.4) |  |
| Aware but not practicing | 25 (11.4) | 67 (18.0) | 86 (28.9) |  |
| Aware and practicing | 35 (16.0) | 88 (23.7) | 50 (16.8) |  |
| Knowledge accuracy | 190 (87.9) | 141 (38.4) | 64 (22.5) | 0.001 |
|  |  |  |  |  |
| **Sleep guidelines** |  |  |  | 0.163 |
| Not aware | 65 (29.8) | 109 (29.4) | 100 (33.8) |  |
| Aware but not practicing | 38 (17.4) | 88 (23.7) | 51 (17.2) |  |
| Aware and practicing | 115 (52.8) | 174 (46.9) | 145 (49.0) |  |
| Knowledge accuracy | 125 (58.9) | 257 (71.6) | 195 (67.9) | 0.008 |
| **Screentime guidelines** |  |  |  | 0.001 |
| Not aware | 111 (50.7) | 176 (47.6) | 145 (49.0) |  |
| Aware but not practicing | 22 (10.1) | 89 (24.1) | 79 (26.7) |  |
| Aware and practicing | 86 (39.3) | 105 (28.4) | 72 (24.3) |  |
| Knowledge accuracy | 59 (27.1) | 94 (26.2) | 98 (33.6) | 0.09 |

Missing data: Physical activity guidelines - Toddler (n=7), Pre-schooler (n=5); knowledge estimates for physical activity recommendations - Infant (n=3), toddler (n=12), pre-schoolers (n=18) ; Sleep guidelines- Infant (n=1) , toddler (n=8), pre-schoolers (n=7) ; Knowledge estimates for sleep recommendations - Toddler (n=98), Pre-schooler (n=10) ; Screen viewing time guidelines - (Toddler n=9), Pre-schooler (n=7); knowledge estimates for screen viewing time recommendations (n=1), Toddlers (n=9), Pre-schoolers (n=11)
